# Improved Clinical Outcomes of High Risk β Thalassemia Major Patients Undergoing a HLA Matched Related Allogeneic Stem Cell Transplant with a Treosulfan Based Conditioning Regimen and Peripheral Blood Stem Cell Grafts

**DOI:** 10.1371/journal.pone.0061637

**Published:** 2013-04-26

**Authors:** Vikram Mathews, Biju George, Auro Viswabandya, Aby Abraham, Rayaz Ahmed, Abhijeet Ganapule, Eunice Sindhuvi, Kavitha M. Lakshmi, Alok Srivastava

**Affiliations:** Department of Haematology, Christian Medical College, Vellore, India; Emory University/Georgia Insititute of Technology, United States of America

## Abstract

Improving clinical outcomes among high risk Class III β thalassemia major patients (Class IIIHR) receiving an allogeneic SCT remains a challenge. From October, 2009 a treosulfan based regimen (TreoFluT) was used for all consecutive Class III patients (n = 50). The clinical outcomes were compared with the historical conventional busulfan (BuCy) based regimen (n = 139). Use of TreoFluT was associated with a significantly reduced incidence of sinusoidal obstruction syndrome (SOS) among Class IIIHR cases (78% to 30%; P = 0.000) and early TRM (46% to 13%; p = 0.005). There was also a trend towards better engraftment in the Class IIIHR subset (P = 0.055). However, the use of bone marrow (BM) as source of stem cells along with the TreoFluT regimen was associated with 50% early mixed chimerism which reduced to 8.5% with the use of a peripheral blood stem cell graft (PBSC). Use of a PBSC graft was not associated with a significant increase in the incidence of acute or chronic graft versus host disease (GVHD). The overall and event free survival was significantly better among the Class IIIHR subset with the use of TreoFluT Vs. BuCy (86.6±7.3 Vs. 39.4±6.8%; P = 0.002 and 77.8±8.8 Vs. 32.4±6.5%; P = 0.003 respectively). A TreoFluT conditioning regimen with a PBSC graft can significantly improve clinical outcomes of Class IIIHR patients.

## Introduction

Allogeneic stem cell transplantation (SCT) remains the only curative option for patients with β thalassemia major. The correction of this disorder by an allogeneic stem cell transplant was first described by Thomas et al [1]. Subsequently, a myeloablative conditioning regimen of busulfan and cyclophosphamide was established and has been the standard of care for stem cell transplantation in this condition [2]. The current risk stratification [3], [4] of patients with β thalassemia major undergoing a myeloablative allogeneic stem cell transplantation (SCT) has significant limitations and fails to recognize a very high risk group. Prior to stem cell transplant patients who were ≥7 years old and had a liver size ≥5 cms constitute what we previously defined as a very high risk subset of a conventional high risk Class III group (Class III high risk or Class IIIHR) [5]. The adverse impact of age and liver size was further validated by an international collaborative analysis which was recently reported [6]. Class III and more specifically Class III high risk (HR) subset have a high risk of graft rejection and regimen related toxicity especially sinusoidal obstruction syndrome (SOS) leading to multi-organ failure and death. The poor clinical outcome in this subset of older patients with very poor pre-transplant medical therapy, as reported by us, is not reflected in the Western literature due to the use of adequate blood transfusion and chelation support prior to transplant. However, when such a population is transplanted even in a developed country with expertise in such transplants the rejection rate is as high as 34% [7].

This has lead to the evaluation of a number of novel conditioning regimens in this group [3], [4], [8], [9], [10], [11]. Treosulfan (dihydroxybusulfan), in the recent past, has attracted a lot of attention as an agent to replace busulfan in view of its favorable toxicity profile [12]. It is especially attractive in the context of an allogeneic SCT for high risk β thalassemia major because of its reported low hepatic toxicity profile and consistent pharmacokinetic profile which are both significant problems with conventional busulfan in this population [5], [13], [14]. Bone marrow has been the preferred choice of stem cells to reduce the risk of graft versus host disease (GVHD) in this non malignant condition, though the incidence of both acute and chronic GVHD in this predominantly pediatric population is low [14], [15]. Recently a similar low incidence of acute and chronic GVHD with a PBSC graft in the setting of matched unrelated transplants has been reported from China [16].

We report our experience of allogeneic SCT in Class III and the Class IIIHR subset and the impact of the use of a previously reported reduced toxicity Treosulfan based regimen [11], [17] modified by the use of peripheral blood stem cell (PBSC) graft.

## Materials and Methods

This is a retrospective analysis of anonymous patient data. All data was taken from patient records at the Christian Medical College and Hospital, and this study was approved by the ethical review board of Christian Medical College.

All consecutive transfusion dependant β thalassemia patients with a HLA identical related donor or a ≥9 of 10 high resolution HLA matched unrelated donor who underwent an allogeneic SCT at our centre from October, 1991 to February, 2012 were included in this analysis.All patents and donors underwent the procedure after getting written and informed consent. The consent forms and consent procedure was approved by the institutions review board. Hard and soft copies of the consent form and all the data generated from the transplant procedure on patients and donors is filed permanently in our department.

### Pre-transplant evaluation

All patients were evaluated with a complete blood count (CBC), biochemical profile and serology for HIV, HBV, HCV and CMV. Pre-transplant a liver biopsy was performed at the time of Hickman catheter insertion.

### Conditioning

Conditioning regimensin the majority was a myeloablative regimen consisting of a combination of busulfan and cyclophosphamide (BuCy) as previously reported by us [14]. From October, 2009 till to date, in an effort to reduce the treatment related morality (TRM) and graft rejection in our high risk cases, the conditioning regimen was modified. This involved the use of a Treosulfan based conditioning regimen as previously reported [11], [17]and consisted of administering thiotepa 8 mg/kg on day -6, fludarabaine 30 mg/m^2^/day×4 days from day-5 to -2 and treosulfan 14 gm/m^2^×3 days from day-5 to -3 (TreoFluT).

### Bone Marrow Harvest

Until the introduction of a TreoFluT conditioning regimen in October 2009 the stem cell source was bone marrow (BM) alone for the majority of patients (98.7%). Bone marrow was harvested under general anesthesia from the iliac crest and the target cell dose was 6×10^8^ nucleated cells/Kg body weight of the recipient. The harvest was collected in ACD with preservative free heparin using one-liter harvest bags. For major ABO mismatched transplants, red cell depletion was achieved with 6% hydroxyethyl starch and gravity sedimentation. After the introduction of the treosulfan conditioning regimen in October, 2009 the stem cell source initially continued to be BM until October, 2010. Due to increased concerns of early mixed chimerism and graft rejection the stem cell source was changed PBSC for all Class III patients who were conditioned with a TreoFluT based regimenfrom November, 2010. The stem cell products were administered soon after harvest or processing.

### GVHD prophylaxis

Cyclosporine and short course methotrexate was used as GVHD prophylaxis; cyclosporine was administered at a dose of 2.5 mg/Kg intravenously over four hours twice daily starting on day –4 and changed to oral administration at 5 mg/Kg twice daily when mucositis had resolved. Cyclosporine levels were monitored and the dose adjusted to achieve a target level of 100–300 ng/ml. The methotrexate dose was 10 mg/m^2^ on day +1 and 7 mg/m^2^ on day 3, 6 and 11. Acute GVHD was treated with dexamethasone or methyl prednisone. Steroid refractory GVHD was treated as per the discretion of the treating physician.

### Chimerism Analysis

Analysis of chimerism and quantification of recipient and donor in samples was done by polymerase chain reactionamplification of informative STR or VNTR followed by gene scan analysis as has been previously reported by us [18].

### Supportive care

All patients were nursed in a positive pressure HEPA filtered transplant unit. None of the patients received prophylactic antibiotics or underwent gut decontamination. Prophylactic acyclovir was administered for the first 100 days; it was continued beyond day 100 if patient had GVHD and required additional immuno-suppression. Trimethoprim-sulphamethoxazole and oral penicillin prophylaxis was initiated after stable engraftment and continued for a year. At the end of one year all patients who did not have evidence of GVHD and were off all immunosuppressive drugs were vaccinated against polio, diphtheria, tetanus, hemophilus influenza and pneumococcus.

### Definitions

Incidence and severity of GVHD was defined as per established criteria [19]. Hepatic sinusoidal obstruction syndrome (SOS) was diagnosed according to Baltimore criteria [20]. Mixed chimerism for the purpose of this study was defined as ≤95% donor cell percentage. Event free survival (EFS) was defined from the time of transplant to an event; an event was primary graft rejection, death or recurrence of transfusion dependence. Stable mixed chimerism with transfusion independence was not considered an event for this analysis. Overall survival (OS) was defined as time from transplant to death due to any cause.

### Statistical analysis

The χ2, t test or fisher exact test and Mann Whitney U test were used to compare differences between groups. Probabilities of event free survival (EFS) and overall survival (OS) following transplant were estimated by the Kaplan-Meier method and the significance assessed by log-rank test. The relationships of clinical features to outcome were analyzed by Cox proportional hazard model.Significance levels were set at 0.05. Statistical analysis was done using SPSS version 11.0.

## Results

### Demographic and Clinical Characteristics

A total of 362 patients with transfusion dependent β thalassemia underwent an allogeneic SCT between January, 1991 and February, 2012 at our centre. 358 (98.8%) of these transplants were from related donors and of the related donors 348 (97.2%) were HLA identical. The median age of this cohort was 7±4.4 years and there were 227 males (62.7%). There were 16(4.4%), 144 (39.8%) and 202 (55.8%) transplants in Lucarelli Class I, II and III respectively.

Of the 202 Class III patients, 82 (40.5%) were classified as Class IIIHR (Age≥7 years and liver size≥5 cms). The conditioning regimen used for the Class III patients was BuCy in 139 (68.8%) and TreoFluT in 50 (24.7%). Similarly, the 82 cases identified as Class IIIHR were conditioned with BuCy in 54 (65.8%) and TreoFluT in 24 (29.2%). The baseline characteristics and clinical outcomes of Class III and Class IIIHR patients conditioned with BuCy and TreoFluTregimens are summarized in Tables 1 and 2 respectively.With the exception of the significantly higher use of PBSC grafts among those conditioned with the TreoFluT regimen there were no other significant differences in baseline characteristics among patients conditioned with these regimens among the entire Class III (Table 1) and the Class IIIHR subset (Table 2).

**Table 1:** Baseline characteristics, supportive care and clinical outcomes of Class III patients receiving different conditioning regimens.

Variable	Bu/Cy^a^ (n = 139) n(%)/Median (range)/Mean ± SD	TreoFluT^b^ (n = 50) n(%)/Median (range)/Mean ± SD	P-value
Age	9 (2–24)	11 (2–21)	0.108
Sex: Male	87 (63)	30 (60)	0.738
Female donor to Male recipient (F>M)	50 (36)	14 (28)	0.384
HBsAg positive	5 (4)	1 (2)	1.000
HCV positive	28 (20)	7 (14)	0.401
CMV IgG positive	130 (96)	47 (94)	0.448
Liver Size (cms)	4.6±1.7	4.6±1.8	0.907
Splenectomy	30 (22)	8 (16)	0.537
ALT^*^ (IU/Lt)	80 (10–357)	54 (8–230)	0.031
AST^∧^ (IU/Lt)	56 (15–199)	45 (14–186)	0.143
Serum Ferritin (ng/ml)	4096 (681–13600)	4380 (1449–10950)	0.397
Number of packed cells transfused pre-transplant	120 (20–450)	119.5 (22–280)	0.653
Stem cell source:			
BM^@^	128 (92)	13 (26)	
G-CSF primed BM	9 (7)	-	0.000
PBSC^#^	2 (1)	37 (74)	
CD34 (×10^6^/kg)	6.7 (2.64–15.9)	11.4 (2–30)	0.000
Engraftment	122 (88)	47 (94)	0.289
Engraftment (days):			
Time to ANC>0.5×10^9^/Lt	16.9±4.1	16.1±3.2	0.242
Time to platelet>20×10^9^/Lt	32.7±17.6	18.8±8.0	0.000
Peri-transplant support:			
(up to day 100)			
Platelet units transfused	23 (5–92)	13 (1–76)	0.000
PRCθ units transfused	19 (0–357)	13 (1–91)	0.003
Sinusoidal obstruction syndrome	92 (66)	11 (22)	0.00
Graft Rejection	16 (12)	4 (8)	0.599
Graft versus host disease:			
Acute GVHD^©^	55 (44)	17 (35)	0.305
Chronic GVHD^©^	17 (18)	4 (11)	0.430
Treatment related mortality(<day 100)	39 (28)	6 (12)	0.021
Overall Survival 3 yrs∞	63.6±4.2	87.4±4.8	0.011
Event free survival 3 yrs∞	57.3±4.3	78.8±6.0	0.041

a BuCy: Busulfan + Cyclophosphamide; *ALT: Alanine aminotransferase.

b TreoFluT: Treosulfan, fludarabine and thiotepa; ∧AST: Aspartate aminotransferase.

@ BM: Bone marrow; #PBSC: Peripheral blood stem cells.

θ PRC: Packed red cell units; ^©^GVHD: graft versus host disease.

∞ 3 yrs. 3 years Kaplan-Meier estimate±1SE%.

**Table 2:** Baseline characteristics, supportive care and clinical outcomes of Class IIIHR (High Risk) group receiving different conditioning regimens.

Variable	Bu/Cy^a^(n = 54) n(%)/Median (range)/Mean ± SD	TreoFluT^b^ (n = 24) n(%)/Median (range)/Mean ± SD	P-value
Age	12 (7–24)	12 (3–21)	0.565
Sex: Male	31 (57)	16 (67)	0.466
Female donor to Male recipient (F>M)	19 (35)	5 (21)	0.289
HBsAg positive	3 (6)	-	0.549
HCV positive	11 (20)	5 (21)	1.000
CMV IgG positive	52 (96)	22 (92)	0.097
Liver Size (cms)	6.2±1.4	6.1±1.2	0.770
Splenectomy	22 (41)	7 (29)	0.448
ALT^*^(IU/Lt)	106 (20–309)	49 (8–230)	0.008
AST^∧^ (IU/Lt)	65 (18–199)	43.5 (14–186)	0.048
Serum Ferritin (ng/ml)	4537 (1528–11885)	4591 (1838–10710)	0.799
Number of packed cells transfused pre-transplant	152 (72–450)	150 (26–280)	0.422
Stem cell source:			
BM^@^	52 (96)	7 (29)	
G-CSF primed BM	2 (4)	-	0.000
PBSC^#^	-	17 (71)	
CD34 (×10^6^/kg)	5.8 (2.9–14.8)	9.95 (2–30)	0.001
Engraftment	42 (78)	23 (96)	0.055
Engraftment (days):			
Time to ANC>0.5×10^9^/Lt	17.0±5.1	15.8±3.4	0.503
Time to platelet>20×10^9^/Lt	35.4±12.9	20±10.2	0.000
Peri-transplant support:			
(upto day 100)			
Platelet units transfused	21 (5–92)	16.5 (1–76)	0.063
PRCθ cell units transfused	18 (5–357)	13.5 (1–88)	0.020
Sinusoidal obstruction syndrome	42 (78)	7 (30)	0.000
Graft Rejection	7 (13)	2 (8)	0.713
Graft versus host disease:			
Acute GVHD^©^	23 (55)	8 (35)	0.194
Chronic GVHD^©^	6 (21)	2 (11)	0.445
Treatment related mortality(<day 100)	25 (46)	3 (13)	0.005
Overall Survival 3 yrs∞	39.4±6.8	86.6±7.3	0.002
Event free survival 3 yrs∞	32.4±6.5	77.8±8.8	0.003

a BuCy: Busulfan + Cyclophosphamide; *ALT: Alanine aminotransferase.

b TreoFluT: Treosulfan, fludarabine and thiotepa; ∧AST: Aspartate aminotransferase.

@ BM: Bone marrow; #PBSC:Peripheral blood stem cells.

θ PRC: Packed red cell units; ^©^GVHD: graft versus host disease.

∞ 3 yrs. 3 years Kaplan-Meier estimate±1SE%.

### Engraftment and day28 chimerism analysis

The proportion of patients who engrafted post-transplant was higher with the TreoFluT based conditioning regimen (Table 1) and this tended to statistical significance in the Class IIIHR subset (Table 2: 78% Vs. 96% in favor of the TreoFluT group; P-value0.055). The time to achieve a platelet count >20×10^9^/Lt was significantly shorter with the TreoFluT conditioning regimen for the entire Class III and the Class IIIHR subset (Table 1 and 2). This was predominantly contributed by the use of a PBSC graft in patients conditioned with TreoFluT as illustrated in Table 3. This faster engraftment translated into significantly lower number of platelet and packed red cell units (PRC) transfused in the peri-transplant period (Table 1). Among patients conditioned with a TreoFluT regimen the use of PBSC grafts was also associated with significant reduction in peri-transplant platelet and PRC units transfusedin comparison to those that received a BM graft (Table 3).

**Table 3:** Comparison of Class III patients who received TreoFluTconditioning with either a bone marrow or peripheral blood source of stem cells.

Variable	BM@ (n = 13) n(%)/Median range)/Mean ± SD	PBSC^#^ (n = 37) n(%)/Median (range)/Mean ± SD	P-value
Age	9 (6–14)	11 (2–21)	0.001
Sex: Male	10 (77)	20 (54)	0.197
Female donor to Male recipient (F>M)	4 (31)	10 (27)	1.000
CD34 (×10^6^/kg)	8.8 (2.1–24.4)	12.5 (2–30)	0.000
Engraftment	12 (92)	35 (95)	1.000
Engraftment (days):			
Time to ANC>0.5×10^9^/Lt	16.4±2.6	16.0±3.4	0.051
Time to platelet>20×10^9^/Lt	26.2±8.8	16.5±6.3	0.000
Chimerism:			
Evaluated on Day 28:	12 (92.3)	35 (94.5)	0.720
Percentage donor cells	97.5 (52–100)	100 (0–100)	0.004
Mixed chimerism	6 (50%)	3 (8.5%)	0.005
Graft Rejection	2 (15)	2 (5)	0.275
Peri-transplant support:			
(upto day 100)			
Platelet units transfused	20 (10–31)	13 (1–76)	0.000
PRCθ units transfused	17 (8–65)	12 (1–91)	0.001
Sinusoidal obstruction syndrome	5 (39)	6 (17)	0.133
Graft versus host disease			0.498
Acute GVHD^©^	3 (23)	14 (39)	1.000
Chronic GVHD^©^	1 (9)	3 (12)	
Treatment related mortality(<day 100)	2 (15)	4 (11)	0.643
Overall Survival 3 yrs∞	72.2±2.7	87.3±5.4	0.717
Event free survival 3 yrs∞	65.9±2.8	73.9±11.2	0.309

@ BM: Bone marrow; #PBSC: Peripheral blood stem cells.

θ PRC: Packed red cell units; ^©^GVHD: graft versus host disease.

∞ 3 yrs. 3 years Kaplan-Meier estimate±1SE%3.

**Figure 1:**
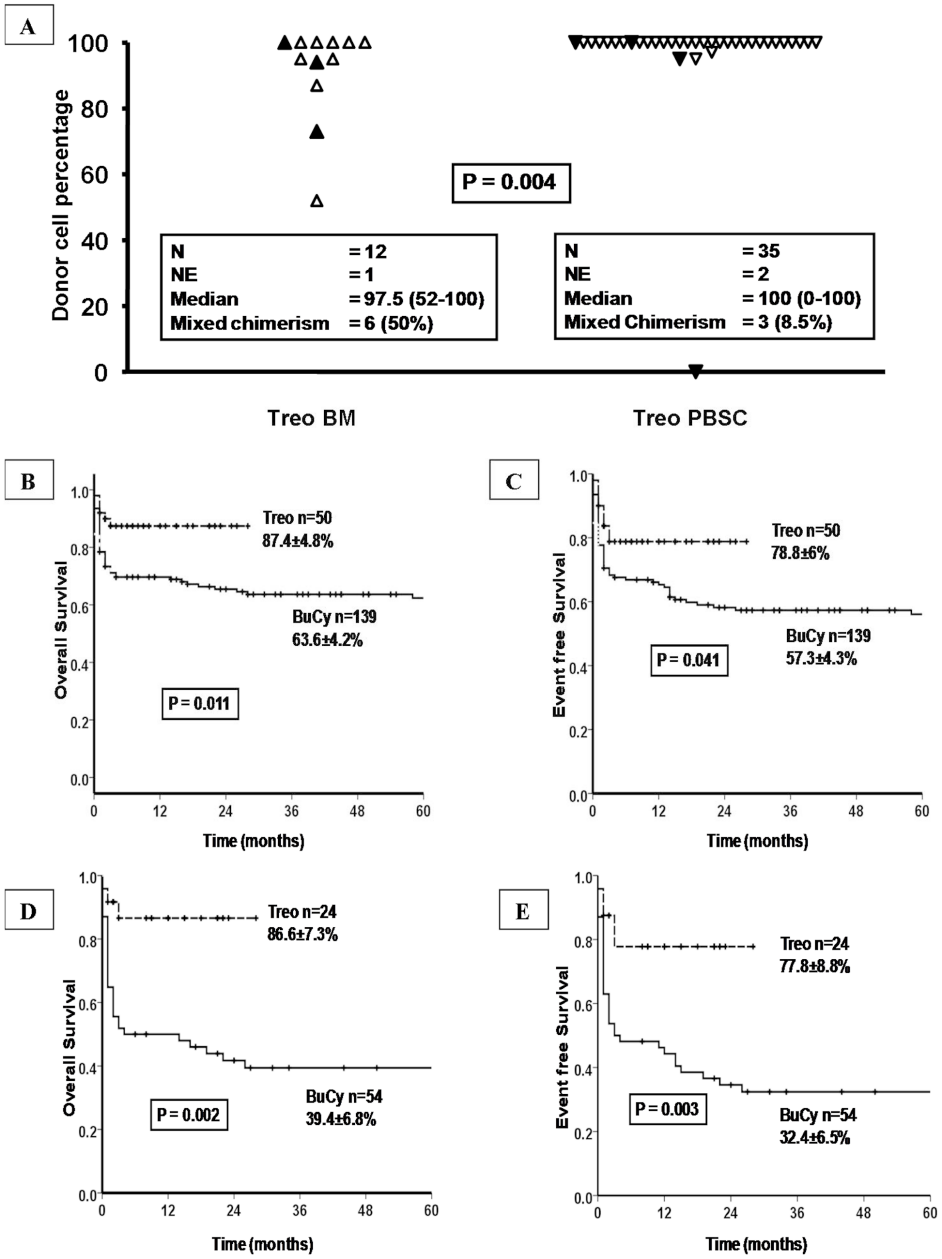
Analysis of day 28 chimerism and survival in different subsets. (A) Day 28 chimerism comparing patients conditioned with aTreoFluT regimen and receivingeither a bone marrow graft (Treo BM) or a peripheral blood stem cell graft (Treo PBSC); NE = not evaluated; indicates cases that had died prior to day 28 and were hence: not evaluated. ▴ Filled triangles indicates cases that had an event defined as either graft rejection or death. △ Empty triangles indicate cases that did not have an event defined as either graft rejection or death (B) Overall survival of all Class III patients conditioned with either a TreoFluT (Treo) or a busulfan based regimen (Bu/Cy) (C) Event free survival of all Class III patients conditioned with either a TreoFluT (Treo) or a busulfan based regimen (Bu/Cy) (D) Overall survival of all Class IIIHR patients conditioned with either a TreoFluT (Treo) or a busulfan based regimen (Bu/Cy) (E) Event free survival of all Class IIIHR patients conditioned with either a TreoFluT (Treo) or a busulfan based regimen (Bu/Cy).

The early experience with the TreoFluT regimen using a BM graft (n = 13) resulted in 6 (50%) of evaluated patients (one died prior to day 28) having mixed chimerism. For recipients with mixed chimerism intervention with rapid withdrawal of cyclosporine and donor lymphocyte infusion was considered when serial chimerism values showed a downward trend with no evidence of graft versus host disease (GVHD) or if the recipient became transfusion dependent in the absence of evidence of post-transplant pure red cell aplasia. Of the 6 cases with mixed chimerism 2 went on to complete rejection (day 74 and day 76) while 4 were salvaged with these interventions (2 received DLI). Following this experience the graft source was changed to PBSC for Class III patients conditioned with TreoFluT. With the use of PBSC (n = 37), mixed chimerism was seen in 3 (8.5%) of the 35 patients evaluated (2 died prior to day 28). Of these three, one patient was a 20 year old man who underwent a 9/10 MUD transplant. This patient had a documented parvovirus B19 infection on day 15 and went on to have a primary graft failure with autologous recovery. The remaining two cases were salvaged with rapid tapering of immunosuppression and infusion of DLI. Figure 1A summarizes the day 28 chimerism data among patients receiving a TreoFluT conditioning regimen. The number of cases with mixed chimerism was higher and the median day 28 chimerism was significantly lower in patients receiving a BM graft (Figure 1A).

### Sinusoidal obstruction syndrome

There was significant reduction in incidence of SOS in Class III patients (Table 1: P-value = 0.000) and Class IIIHR patients (Table 2: P-value = 0.000)conditioned with a TreoFluTregimen. Among Class IIIHR cases, those conditioned with BuCy had a 78% incidence of SOS while those conditioned with the TreoFluT regimen it was 30%. Of the 7(30%) Class IIIHR cases conditioned with TreoFluT that had a documented SOS, it was mild self-limiting and managed conservatively in 6 and in only one case did it result in multi-organ failure leading to death. In contrast among Class IIIHR cases conditioned with a BuCy regimen in 10 (24%) of the 42 cases documented to have SOS this was the major contributory cause of death. The low incidence of SOS and other regimen related toxicity translated to a significantly lower day 100 treatment related mortality (TRM) among Class IIIHR patient conditioned with TreoFluT (Table 2: 13% Vs. 46%; P-value-0.005).

### Graft versus host disease

There was no significant difference in the incidence of acute or chronic GVHD among Class III or Class IIIHR cases conditioned with either regimens (Table 1 and 2 respectively). Among Class III patient's conditioned with a TreoFluT regimen who received either a BM or a PBSC graft Grade 2–4 acute GVHD was seen in 3 (23%) Vs. 10 (27%)respectively while Grade 3–4 acute GVHD was seen in none Vs. 3 (8.1%) respectively (Table 3). Of the 3 patients who developed Grade 3–4 acute GVHD among those who received a PBSC graft, one patient responded to therapy and has complete resolution of GVHD while the other two succumbed to it.Among the 3 (12%) that developed chronic GVHD after a TreoFluT conditioning regimen and PBSC graft(Table 3) it was extensive in one while it was limited in the other two.

### Survival

After excluding patients who had early TRM (<100 days) the actuarial median follow up of this cohort was 42 months (range: 3–254). The 3 year Kaplan-Meier estimate of OS and EFS of the entire Class III cohort was 67.4±3.5% and 58.9±3.7% respectively. Among the Class III patients use of a TreoFluT regimen was associated with a significant improvement in OS and EFS (as illustrated in Figure 1C and 1D) respectively. Similarly, the OS and EFS were significantly superior in the Class IIIHR subset that was conditioned with a TreoFluT regimen (illustrated in Figure 1E and 1F respectively).

### Cause of deaths

Among the Class III patients 39 (28%) of the cases conditioned with a Bu/Cy regimen died while 6 (12%) of those conditioned with a TreoFluT regimen died. Table 4 summarizes the major contributory cause of death among these two groups. As illustrated in table 4, SOS was a major contributing factor for death when Bu/Cy was the conditioning regimenand was significantly lower when a TreoFluT regimen was used. Deaths beyond day 100 was seen in 12 (8.6%) of cases conditioned with Bu/Cy and the major contributory cause was infections. Among those conditioned with TreoFluT there were 2 (4%) deaths beyond day 100, both were related to graft rejection and both had received a BM graft.

**Table 4:** Comparison of major contributory cause of early death (death <100 days post transplant) among Class III patients.

Variable	Bu/Cy^a^ n = 139	Treo^b^ n = 50	P-value
Died < day 100	39 (28%)	6 (12%)	0.001
Rejection	5 (3.6%)	-	0.581
Sinusoidal obstruction syndrome	17 (12.1%)	1 (2%)	0.035
Infection	7 (5%)	2 (4%)	0.768
DAH#	2 (1.4%)	-	0.394
Cardiac failure	2 (1.4%)	1 (2%)	0.798
Hemorrhagic cystitis	1 (0.7%)	-	0.548
GVHD©	2 (1.4%)	2 (4%)	0.281
Intra-cerebral bleed	3 (2.1%)	-	0.295

a BuCy Busulfan + Cyclophosphamide.

b TreoFluT Treosulfan, fludarabine and thiotepa.

# DAH Diffuse alveolar hemorrhage.

© GVHD Graft versus host disease.

## Discussion

Improving clinical outcomes of Class III and the Class IIIHR [5] β thalassemia major following an allogeneic SCT remains a challenge. We had previously reported the poor clinical outcome of our newly recognized Class IIIHR subset following allogeneic SCT using a conventional myeloablative Bu/Cy regimen [5].

The major contributory factors for the poor clinical outcome in these patients were a combination of high risk for graft rejection and regimen related toxicity (RRT) [5]. Early attempts to reduce RRT in a similar cohort of cases were done by reducing the cumulative dose of busulfan and cyclophosphamide. Experience from such an approach, as reported by the Pesaro group, suggested that a reduction in TRM was achieved but there was a significantly higher risk of graft rejection [10]. While other regimens have been reported to improve the clinical outcome of Class III thalassemia patients undergoing an allogeneic SCT [9] we would argue that most published data does not address the very high risk subset as defined by us [5]. The clinical outcome of a similar cohort reported from a developed country center would suggest that one could anticipate a graft rejection close to 34% [7] with intravenous busulfan in spite of pharmacokinetic monitoring and appropriate dose modifications.

We were particularly attracted to the treosulfan based regimen due to the low hepatotoxicity profile of this agent [12] and the reported low incidence of SOS in patients with thallasemia major [11], [17]. Hepatic SOS was a major problem with a conventional myeloablative regimen in our Class IIIHR patients with 78% of cases developing this complication and in 24% of such cases it lead to multi-organ failure and death.

Our initial experience with this TreoFluT regimen with a BM graft in this cohort at a very high risk for graft rejection was concerning, with 6 (50%) having early mixed chimerism. Our experience and reported data would suggest that early mixed chimerism, unlike late stable mixed chimerism, usually predicts secondary graft rejection in up to 50% of these cases [21]. Four of these initial 6 cases of mixed chimerism were salvaged with rapid taper of immune-suppression (2 of these required additional DLI to restore transfusion independence). The regimen itself was very well tolerated. We then opted to use a PBSC graft source with the same conditioning regimen. This approach resulted in greater consistency of early complete chimerism and was associated with additional benefits such as faster engraftment and significantly reduced peri-transplant transfusion support. Use of a PBSC graft did not result in a significantly higher incidence of acute or chronic GVHD compared to a BM graft with the same regimen. Chronic GVHD remains a concern with the use of a PBSC graft and this is one of the reasons why we have restricted the use of this regimen along with a PBSC graft to Class III patients.

Unfortunately, in resource constrained environments, such as ours and many other developing countries, it is not uncommon for a majority of patients with β thalassemia major to present late for transplantation. The body mass index (BMI) of our Class III patients is reflective of the poor clinical condition of a majority of these patients with the median BMI being 14.9 (range: 10.2–23.8) (figure S1). Only 22 (10.9%) of our Class III patients had a BMI in the normal range.

This retrospective data analysis is limited by the comparison of the use of TreoFluT regimen with a historical control spanning more than two decades. However, as illustrated in figure S2 the clinical outcome analyzed by 5 year intervals since the start of our transplant program reflects that there has been marginal, statistically not significant, improvement in the event free survival of our Class III or Class IIIHR cases over this time period. This illustrates that improvements in techniques and supportive care cannot explain the improved outcome seen with this treosulfan based regimen in our center.

In summary, this data suggests that even in a very high risk group of β thalassemia major patients undergoing an allogeneic SCT the benefits of this TreoFluT regimen along with a PBSC graft outweighs the risks and can significantly improve the overall and event free survival.

## Supporting Information

Figure S1 **Body mass index of Class III patients.**
(DOCX)

Figure S2 **Event free survival of (A) Class III and (B) Class IIIHR over different time periods of allogeneic stem cell transplantation at our center.**
(DOCX)
