# Supplementary material for: Improved Clinical Outcomes of High Risk β Thalassemia Major Patients Undergoing a HLA Matched Related Allogeneic Stem Cell Transplant with a Treosulfan Based Conditioning Regimen and Peripheral Blood Stem Cell Grafts
Source: PLoS One. 2013 Apr 26;8(4):e61637. doi: 10.1371/journal.pone.0061637 (PMC3637210; doi:10.1371/journal.pone.0061637)
Supplement: Figure S1 — Body mass index of Class III patients. (DOCX) [file pone.0061637.s001.docx]

Supplementary Data

Figure S1: Body mass index of Class III patients.

n = 202

Median = 14.9 (range: 10.2 – 23.8)

The WHO criteria for very severely under weight is BMI <15.

Number of patients with normal BMI = 22 (10.9%)

**P=0.818**
