# Supplementary material for: Improved Clinical Outcomes of High Risk β Thalassemia Major Patients Undergoing a HLA Matched Related Allogeneic Stem Cell Transplant with a Treosulfan Based Conditioning Regimen and Peripheral Blood Stem Cell Grafts
Source: PLoS One. 2013 Apr 26;8(4):e61637. doi: 10.1371/journal.pone.0061637 (PMC3637210; doi:10.1371/journal.pone.0061637)
Supplement: Figure S2 — Event free survival of (A) Class III and (B) Class IIIHR over different time periods of allogeneic stem cell transplantation at our center. (DOCX) [file pone.0061637.s002.docx]

Supplementary Data

**Figure 2S**: Event free survival of (A) Class III and (B) Class IIIHR over different time periods of allogeneic stem cell transplantation at our center.

**P=0.613**

**P=0.818**
